# Should Tyrosine Kinase Inhibitors Be Considered for Advanced Non–Small-Cell Lung Cancer Patients With Wild Type EGFR? Two Systematic Reviews and Meta-Analyses of Randomized Trials^☆^

**DOI:** 10.1016/j.cllc.2014.11.007

**Published:** 2015-05

**Authors:** Claire L. Vale, Sarah Burdett, David J. Fisher, Neal Navani, Mahesh K.B. Parmar, Andrew J. Copas, Jayne F. Tierney

**Affiliations:** 1 MRC Clinical Trials Unit at UCL, London, United Kingdom; 2 Department of Thoracic Medicine, University College London Hospital, London, United Kingdom

**Keywords:** Chemotherapy, Meta-analysis, NSCLC, RCT, TKI

## Abstract

Guidance concerning tyrosine kinase inhibitors (TKIs) for patients with wild type epidermal growth factor receptor (EGFR) and advanced non–small-cell lung cancer (NSCLC) after first-line treatment is unclear. We assessed the effect of TKIs as second-line therapy and maintenance therapy after first-line chemotherapy in two systematic reviews and meta-analyses, focusing on patients without EGFR mutations. Systematic searches were completed and data extracted from eligible randomized controlled trials. Three analytical approaches were used to maximize available data. Fourteen trials of second-line treatment (4388 patients) were included. Results showed the effect of TKIs on progression-free survival (PFS) depended on EGFR status (interaction hazard ratio [HR], 2.69; *P* = .004). Chemotherapy benefited patients with wild type EGFR (HR, 1.31; *P* < .0001), TKIs benefited patients with mutations (HR, 0.34; *P* = .0002). Based on 12 trials (85% of randomized patients) the benefits of TKIs on PFS decreased with increasing proportions of patients with wild type EGFR (*P* = .014). Six trials of maintenance therapy (2697 patients) were included. Results showed that although the effect of TKIs on PFS depended on EGFR status (interaction HR, 3.58; *P* < .0001), all benefited from TKIs (wild type EGFR: HR, 0.82; *P* = .01; mutated EGFR: HR, 0.24; *P* < .0001). There was a suggestion that benefits of TKIs on PFS decreased with increasing proportions of patients with wild type EGFR (*P* = .11). Chemotherapy should be standard second-line treatment for patients with advanced NSCLC and wild type EGFR. TKIs might be unsuitable for unselected patients. TKIs appear to benefit all patients compared with no active treatment as maintenance treatment, however, direct comparisons with chemotherapy are needed.

## Introduction

After reports of clinical trials^1-5^ and clinical guidelines (National Institute for Health and Care Excellence [NICE] TA258 and National Comprehensive Cancer Network [NCCN] Non-Small-Cell Lung-17), the use of the tyrosine kinase inhibitors (TKIs), erlotinib and gefitinib, is now common practice for first-line treatment of patients with non–small-cell lung cancer (NSCLC) with sensitizing epidermal growth factor receptor (EGFR) mutations. Beyond first-line treatment, in particular for patients with wild type EGFR who have received first-line chemotherapy, recommendations regarding the potential benefits of TKIs are less clear (NICE TA162, NCCN Guidelines for Non–Small-Cell Lung Cancer).^6,7^

With the exception of a few modern trials that only recruited patients with wild type disease,^8,9^ most evaluations of these drugs have been in unselected patients. Many of these trials did not test for EGFR mutations systematically, therefore, their results are reported either for all randomized patients, ignoring EGFR mutation status, or for the subset of patients in whom the status was known. Results of these trials are mixed and interpretation difficult.

Similarly, attempts have been made to synthesize existing data, to inform clinical guidelines and practice. Some have pooled all available results irrespective of mutation status or treatment line.^10,11^ This approach gives results based on large numbers of randomized patients, but results are difficult to interpret. Others have attempted to summarize the results of trials within subgroups defined according to EGFR status.^12,13^ Although this seems sensible and offers a more meaningful interpretation of the results, there are clear drawbacks. For example, trials that did not report their results according to mutation status were excluded, as were patients for whom no EGFR testing was carried out. When only a subset of the randomized patients within a given trial were tested, pooling results might introduce bias,^14^ not least because we cannot be certain that these patients were representative.

Despite these difficulties, there is a need to obtain effective and reliable summaries of the effects of TKIs in patients with and without sensitizing EGFR mutations after first-line treatment. Therefore, we set out to take into account the potential issues and biases. Rather than relying on one analytical approach, we planned a number of separate analyses. In addition to pooled analyses of all patients from all trials and of patient subgroups defined according to EGFR status, we aimed to carry out appropriate tests of interaction between treatment effects and patient characteristics, namely mutation status, to ascertain whether effects differed between patient groups. Finally, by assuming the likely ratios of patients with wild type and mutated EGFR in trials based on geographical location (East Asia 60:40/rest of world 90:10),^15^ we could assess the effect of increasing proportions of wild type patients on the overall treatment estimates using metaregression. Interpretation of the results was not based on any one of the individual results from the 3 approaches but on the combined results of all 3 approaches, such that we could feel more confident in our interpretation when results from all 3 analyses were complementary. Analyses were carried out in trials of second-line treatment, when TKIs were compared with standard chemotherapy regimens and in the maintenance therapy setting, compared with no active treatment.

## Materials and Methods

All methods were prespecified in 2 registered protocols (CRD42013006449 and CRD42013006251).

Included trials should have randomized patients with advanced NSCLC irrespective of sex, age, histology, ethnicity, smoking history, or EGFR mutational status. Patients should not have received previous TKIs. For the systematic review of second-line treatment, trials should have compared a TKI (erlotinib or gefitinib) versus chemotherapy after first-line chemotherapy. For maintenance treatment, trials should have compared a TKI (erlotinib or gefitinib) versus no TKI after first-line chemotherapy.

Systematic searches^16^ were conducted in MedLine, EMBASE, Cochrane CENTRAL, clinical trials registers (PDQ, ClinicalTrials.gov), and relevant conference proceedings. We also searched reference lists of relevant randomized controlled trials (RCTs) and clinical reviews.

The risk of bias of individual trials was assessed^16,17^ with a low risk of bias being desirable for sequence generation, allocation concealment, and completeness of outcome data reporting. Trials in the maintenance setting should have also been at low risk of bias for blinding.

Progression-free survival (PFS) was the primary outcome, allowing assessment of the effects of immediate TKI versus no immediate TKI without interference from the use of TKIs on progression. Overall survival (OS) was the secondary outcome, accepting this limitation. Data on patient characteristics, including histology, ethnicity, EGFR mutational and smoking status, interventions, and outcomes were extracted from trial reports. When EGFR mutational status of patients was not reported it was estimated based on trial characteristics.

### Statistical Analysis

Because trials did not necessarily test all patients for EGFR mutations and/or report results according to EGFR mutation status, we used 3 analytical approaches to make maximum use of the data available. Results were assessed for consistency and whether, taken together, they established with greater certainty, the effects of TKIs in patients with wild type EGFR. All interpretation was based on the balance of the results across these 3 approaches and not on any of the individual approaches in isolation.

#### Estimating the Interaction Between Treatment Effect and EGFR Mutation Status

When possible, hazard ratio (HR) estimates of effect and associated statistics were either extracted or estimated from the reported analyses^18-20^ according to EGFR mutation status for each trial. These were used to estimate the interaction between treatment effect and EGFR mutational status, calculated as the ratio of the estimated HRs within the EGFR wild type subgroup and the EGFR mutation subgroup for each trial.^14^ Interaction HRs were combined across trials using the fixed-effects inverse-variance model. Heterogeneity was assessed using χ^2^ test and *I*^2^ statistic.^21^ Such interactions are based on a comparison of the treatment effects of the TKIs for patients with wild type and mutated EGFR within trials. Therefore, the interactions might only be calculated for trials in which patients were tested for EGFR mutation status and the HR estimates for the wild type and mutation populations were reported. They cannot be calculated for trials that recruited patients with wild type EGFR exclusively.

#### Estimating the Effects of Treatment in Patients With Wild Type and Mutated EGFR

To estimate the effect of TKI outcomes for patients with wild type and mutated EGFR, using the maximum available data, the trial HRs and associated statistics were combined across trials using the fixed-effects inverse-variance model. Again, we were restricted to patients tested for EGFR mutation status, but could include trials that exclusively recruited patients with wild type EGFR. Heterogeneity was assessed using the χ^2^ test and *I*^2^ statistic^21^ and where identified, the random effects model was applied. The absolute effect on median PFS was calculated by applying the relevant HR to the average control group median PFS, assuming proportional hazards.

#### Estimating the Effects of Treatment According to the Proportion of Patients With Wild Type EGFR

Using metaregression we investigated how increasing proportions of patients with wild type EGFR (considered as a continuous factor) affected treatment effects. All trials reporting overall estimates of effect were included. Estimates of the effect of TKIs on PFS for all randomized patients were extracted or estimated from the reported analyses.^18-20^ When only a small proportion of patients had been tested for mutation status, we assumed that the proportion of patients with wild type EGFR would remain consistent across the whole trial. When no testing of mutation status had been carried out, or reported in a trial, we estimated the proportions of randomized patients having wild type EGFR to be 90% in western trials and 60% in trials of East Asian patients. We then estimated the change in treatment effect with increasing proportions of patients with wild type EGFR.

We also explored whether the TKI used (erlotinib or gefitinib) or the chemotherapy regimen used (docetaxel alone, pemetrexed alone, or docetaxel with pemetrexed) affected the effect of TKIs. Characteristics found to be important were adjusted for in the metaregression and explored using the F ratio.^22^

## Results

**Figure 1:**
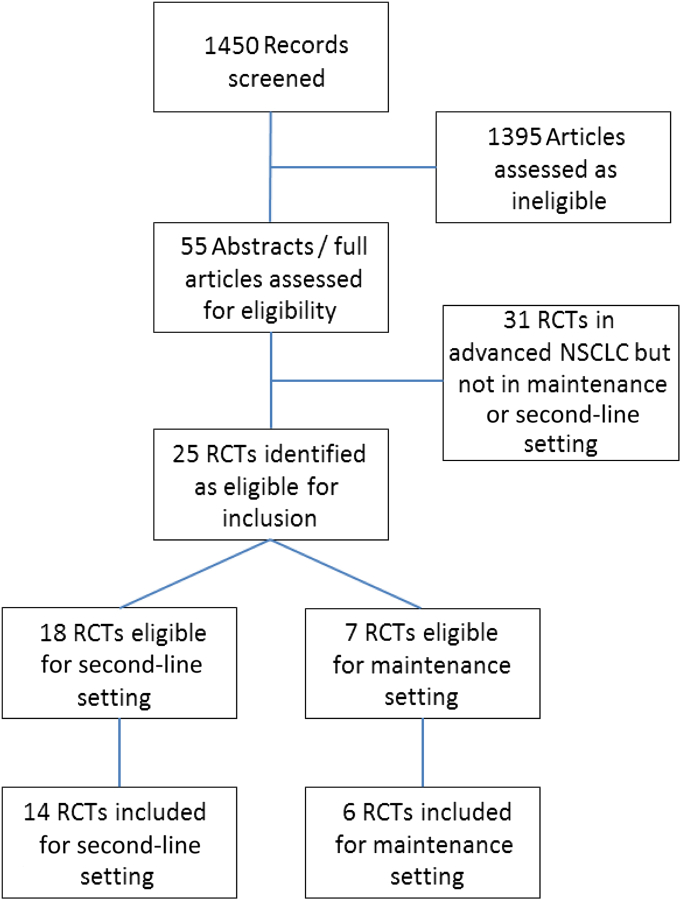
Randomized Controlled Trial (RCT) Identification, Screening, and Inclusion Abbreviation: NSCLC = non–small-cell lung cancer.

We identified 25 potentially eligible RCTs, of TKIs as second-line treatment (n = 18) and maintenance treatment (n = 7; Figure 1).

### Tyrosine Kinase Inhibitor Versus Chemotherapy in the Second-Line Setting

Of 18 potentially eligible trials, randomizing 4456 patients, 1 trial reported no results,^23^ 2 trials^24^ and NCT00536107 were not published, and 1 randomized phase II feasibility trial never reached the phase III stage.^25^ Results were based on the 14 remaining eligible trials (4388 patients, 98% of total randomized, Table 1).^8,9,26-37^ Trials compared TKIs with either docetaxel or pemetrexed chemotherapy and were conducted between 2003 and 2012. Six trials were carried out in predominantly Asian populations. Randomized patients had good performance status (0-2) and median age ranged from 54.5 to 67.5 years (range, 20-88 years). Most were men and either current or former smokers. One trial^33^ included considerably more women (85%) and only never-smokers. Three trials randomized patients with wild type EGFR exclusively.^8,9,37^ Five trials evaluated EGFR mutation status using a range of methods (including DAKO EGFR Pharma DX and Eppendorf Piezo-electric microdissector). Mutation status was not evaluated in 5 trials. Twelve trials (3963 patients, 90% of total) reported PFS and 14 trials (4355 patients, 99% of total) reported OS (Tables 1 and 2).

### Assessment of Risk of Bias

One trial,^36^ published in Chinese language, was judged to be unclear for all domains. The remaining 13 trials were all at low risk of bias regarding incomplete outcome data. Missing data on EGFR mutational status largely resulted from unavailable tumor samples or because the trials were conducted before widespread testing. All were judged to be at low risk of bias for sequence generation. For allocation concealment, 10 trials were judged to be at low risk of bias and 3 were judged as unclear risk. No trials were judged to be at high risk for any of the domains assessed (see Supplemental Table 1 in the online version).^8,9,26-37^ When information could not be obtained from the publications, we contacted the authors.^44^

### Progression-Free Survival

#### Interaction Between Treatment Effect and EGFR Mutation Status

**Figure 2:**
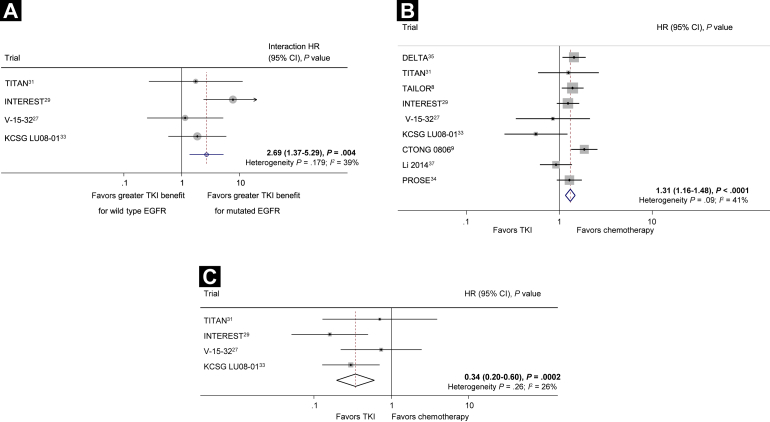
(**A**) Tyrosine Kinase Inhibitor (TKI) Versus Chemotherapy in the Second-Line Setting: Interaction Between Treatment Effect and Epidermal Growth Factor Receptor (EGFR) Mutation Status for Progression-Free Survival. The Circles Represent (Fixed Effect) Meta-Analysis of the Hazard Ratios (HRs) Representing the Interaction Between the Effect of Treatment (TKI) in Wild Type EGFR Compared With Mutated EGFR; the Horizontal Lines Show the 95% CI. (**B**) TKI Versus Chemotherapy in the Second-Line Setting: Effect of Treatment in 1302 Patients With Wild Type EGFR on Progression-Free Survival. Each Square Denotes the HR for That Trial With the Horizontal Lines Showing the 95% CI. The Size of the Square Is Directly Proportional to the Amount of Information Contributed by That Trial. The Diamond Gives the Pooled HR From the Fixed Effect Model; the Center of the Diamond Denotes the HR and the Extremities, the 95% CI. (**C**) TKI Versus Chemotherapy in the Second-Line Setting: Effect of Treatment in 113 Patients With Mutated EGFR on Progression-Free Survival Abbreviations: CTONG = Chinese Thoracic Oncology Group; DELTA = Docetaxel and Erlotinib Lung Cancer Trial; INTEREST = IRESSA Non-small-cell lung cancer Trial Evaluating REsponse and Survival against Taxotere; KCSG = Korean Cancer Study Group; PROSE = Predicting Response to Second-Line Therapy Using Erlotinib; TAILOR = Tarceva Italian Lung Optimization Trial; TITAN = Tarceva In Treatment of Advanced NSCLC.

Data on the effects of TKIs compared with chemotherapy on PFS within groups of patients with EGFR mutations and wild type EGFR were available from 4 trials, including 442 patients with wild type EGFR and 113 with EGFR mutations (13% of the total randomized in all trials). There was strong evidence of an interaction between the effect of TKIs and EGFR mutational status (interaction HR, 2.69; 95% confidence interval [CI], 1.37-5.29; *P* = .004; Figure 2A),^8,9,27,29,31,33,35,37^ with the benefit of treatment of TKIs evident only among patients with EGFR mutations. This was consistent across trials (heterogeneity *P* = .179; *I*^2^, 39%).

#### Effect of Treatment in Patients With Wild-Type and Mutated EGFR

Results for patients with wild type EGFR were available for 9 trials and 1302 patients (30% of the total randomized in all trials). There was evidence of a detriment with TKIs compared with chemotherapy (HR, 1.31; 95% CI, 1.16-1.48; *P* < .0001; Figure 2B), with some evidence of variation between the trial results (heterogeneity *P* = .09; *I*^2^, 41%). However, the effect was fairly similar with a random-effects model (HR, 1.27; 95% CI, 1.08-1.51; *P* = .005). Assuming a median baseline PFS of 13 weeks, based on the average time of PFS in the control arms of included trials; HR, 1.31 translates to a 3-week absolute reduction in median PFS (from 13 weeks to 10 weeks).

Four trials reported PFS for patients with EGFR mutations. Based on these 113 patients (2% of the total randomized in all trials), there was evidence of a benefit with TKIs compared with chemotherapy (HR, 0.34; 95% CI, 0.20-0.60; *P* = .0002) and no evidence of variation between the trial results (heterogeneity *P* = .26; *I*^2^, 26%; Figure 2C). Again, assuming a median PFS of 13 weeks, this translates to a 25-week increase in the absolute median PFS (from 13 weeks to 38 weeks).

#### Effect of Treatment According to Proportion of Patients With Wild Type EGFR

**Figure 3:**
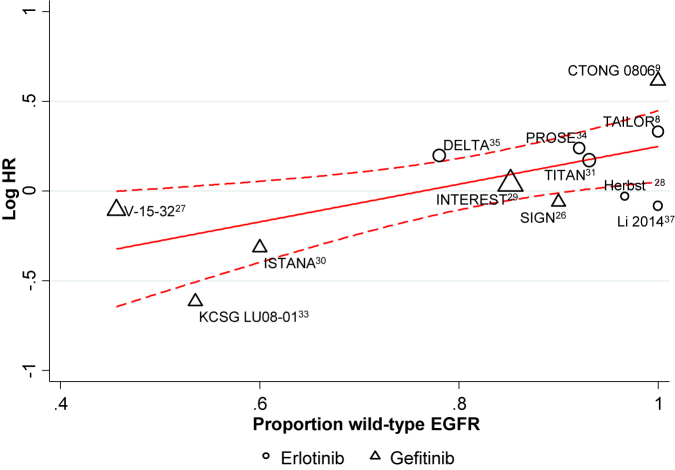
Tyrosine Kinase Inhibitor (TKI) Versus Chemotherapy in the Second-Line Setting: Effect of Treatment According to the Proportion of Patients With Wild-Type Epidermal Growth Factor Receptor (EGFR) on Progression-Free Survival Abbreviations: CTONG = Chinese Thoracic Oncology Group; DELTA = Docetaxel and Erlotinib Lung Cancer Trial; INTEREST = IRESSA Non-small-cell lung cancer Trial Evaluating REsponse and Survival against Taxotere; ISTANA = Iressa as Second-line Therapy in Advanced NSCLC; KCSG = Korean Cancer Study Group; PROSE = Predicting Response to Second-Line Therapy Using Erlotinib; SIGN = Second-line Indication of Gefitinib in NSCLC; TAILOR = Tarceva Italian Lung Optimization Trial; TITAN = Tarceva In Treatment of Advanced NSCLC.

Twelve trials including 3963 patients reported PFS for all patients, irrespective of EGFR status. Metaregression suggested a decreasing effect of TKIs with increasing proportions of wild type patients (*P* = .014). The treatment effect predicted by the model when 100% of patients had wild type EGFR favors chemotherapy (HR, 1.28; 95% CI, 1.08-1.53; *P* = .005), whereas when 100% of patients had EGFR mutations, the model predicted a benefit of TKIs (HR, 0.45; 95% CI, 0.25-0.80; *P* = .007; Figure 3).^8,9,26-29,31,33-35,37^

### Assessing Whether the Treatment Effect Varies With the TKI or Chemotherapy Used

No differences in the treatment effects of TKIs versus chemotherapy were observed when trials were subdivided according to chemotherapy used: docetaxel alone, pemetrexed alone, or docetaxel and pemetrexed (test for between-subgroup heterogeneity *P* = .30). There was a difference in the treatment effect according to the TKI used in all randomized patients (test for between-subgroup heterogeneity *P* = .008). However, when the analysis was adjusted to account for substantial heterogeneity within the group of trials using gefitinib (*P* < .0001; *I*^2^, 82%), there was no longer evidence of this difference between the TKIs (metaregression *P* = .24; F ratio *P* = .18). Additionally, when the TKI type was taken into account in the metaregression, there was still evidence of a decreasing effect of TKIs with increasing proportions of patients with wild type EGFR (*P* = .043).

### Overall Survival

Data on the effects of TKIs on OS within groups of patients with EGFR mutations and wild type EGFR were available from 4 trials, including 540 patients with wild type EGFR and 97 with EGFR mutations (15% of the total randomized in all trials). Based on the available data, there was no evidence of an interaction between the effect of TKIs on OS and EGFR mutational status (interaction HR, 1.15; 95% CI, 0.60-2.18; *P* = .68; Table 2). This relationship appeared consistent across trials (heterogeneity *P* = .37; *I*^2^, 4%).

## Maintenance TKI Versus No Active Treatment

We identified 7 eligible trials. No results were available for 1 ongoing trial (NCT00153803), therefore, 6 trials^38-43^ were included (2697 randomized patients, 100% of total; Table 1). Trials were conducted between 2001 and 2009 and compared TKIs with placebo^38,40-43^ or observation.^39^ Five trials randomized predominantly western patients^38-40,42,43^ and 1 trial randomized only Chinese patients.^41^ Overall, randomized patients had good performance status (0-2); with median age from 55 to 64 years (range, 20-83 years). They were mostly men and either current or former smokers, except for 1 trial,^41^ in which more than half of the included patients had never smoked. Three trials evaluated EGFR mutation status using a range of methods (EGFR mutation detection kit [DxS, Manchester, UK], and sequencing of polymerase chain reaction products from exons 18 to 21 of the *EGFR* gene). Mutation stssatus was not evaluated in the remaining trials.

## Assessment of Risk of Bias

Five trials were judged to be at low risk of bias for allocation concealment, sequence generation, and blinding.^38-41,43^ One trial was at low risk of bias for all domains except for sequence generation and allocation concealment, which were unclear.^42^ No trials were identified as being at high risk of bias. Missing data on EGFR mutational status largely resulted from unavailable tumor samples or because the trials were conducted before widespread testing (see Supplemental Table 1 in the online version).

## Progression-Free Survival

### Interaction Between Treatment Effect and EGFR Mutation Status

**Figure 4:**
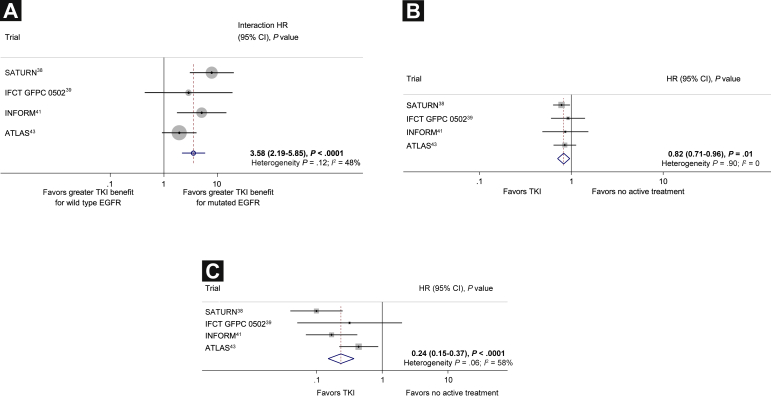
(**A**) Maintenance Tyrosine Kinase Inhibitor (TKI) Versus No Active Treatment: Interaction Between Treatment Effect and Epidermal Growth Factor Receptor (EGFR) Mutation Status for Progression-Free Survival. (**B**) Maintenance TKI Versus No Active Treatment: Effect of Treatment in 778 Patients With Wild Type EGFR on Progression-Free Survival. (**C**) Maintenance TKI Versus No Active Treatment: Effect of Treatment in 130 Patients With Mutated EGFR on Progression-Free Survival Abbreviations: ATLAS = Avastin Tarceva Lung Adenocarcinoma Study; IFCT GFPC = Partenariat Intergroupe Francophone de Cancérologie Thoracique-Groupe Français de Pneumo-Cancérologie; INFORM = Iressa in NSCLC FOR Maintenance; SATURN = Sequential Tarceva in Unresectable NSCLC.

Progression-free survival results were reported separately in 4 trials for wild type patients and EGFR mutation-positive patients, 908 patients (34% of the total randomized in these trials; Table 1). There was strong evidence of an interaction between the effect of TKIs on PFS and EGFR mutational status, with the larger effect being observed in patients with EGFR mutations (interaction HR, 3.58; 95% CI, 2.19-5.85; *P* < .0001; Figure 4A).^38,39,41,43^ There was some evidence of inconsistency in the effect between trials (heterogeneity *P* = .12; *I*^2^, 48%). However, the effect was fairly similar with a random effects model (HR, 3.83; 95% CI, 1.85-7.95; *P* = .0003).

### Effects of Treatment in Patients With Wild Type and Mutated EGFR

Progression-free survival results for patients with wild type EGFR were available from 4 trials and 778 patients. There was evidence of a PFS benefit with TKIs in patients with wild type EGFR (HR, 0.82; 95% CI, 0.71-0.96; *P* = .01; Figure 4B) and no evidence of variation between the trial results (heterogeneity *P* = .90; *I*^2^, 0%). Assuming a median PFS in the control group of 13 weeks, this translates to an absolute improvement in median PFS of approximately 3 weeks (from 13 weeks to 16 weeks).

For patients with EGFR mutations, data were available from 4 trials but only 130 patients. Although the data available for this analysis were very limited, there was a large PFS benefit with TKIs (HR, 0.24; 95% CI, 0.15-0.37; *P* < .0001; Figure 4C) but with clear evidence of variation between the trial results (heterogeneity *P* = .06; *I*^2^, 58%). However, the results were similar when a random effects model was used (HR, 0.22; 95% CI, 0.10-0.46; *P* < .0001). This translated to an absolute improvement in median PFS of approximately 10 months (from 13 weeks to 13 months).

### Effect of Treatment According to the Proportion of Patients With Wild Type EGFR

**Figure 5:**
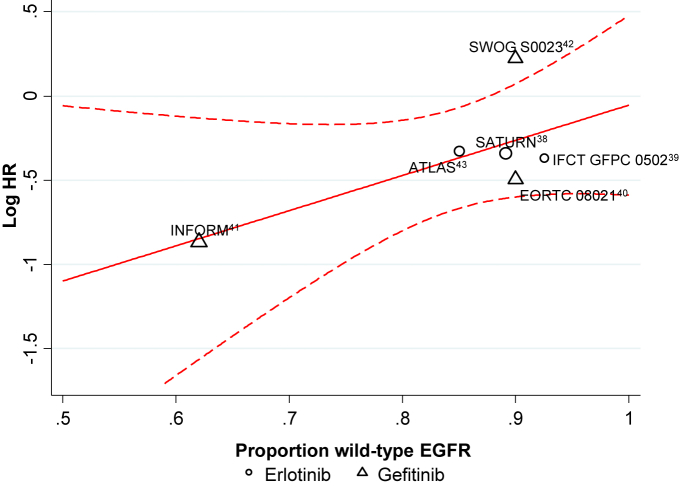
Maintenance Tyrosine Kinase Inhibitor Versus No Active Treatment: Effect of Treatment According to the Proportion of Patients With Wild Type Epidermal Growth Factor Receptor (EGFR) on Progression-Free Survival Abbreviations: ATLAS = Avastin Tarceva Lung Adenocarcinoma Study; EORTC = European Organisation for Research and Treatment of Cancer; IFCT GFPC = Partenariat Intergroupe Francophone de Cancérologie Thoracique-Groupe Français de Pneumo-Cancérologie; INFORM = Iressa in NSCLC FOR Maintenance; SATURN = Sequential Tarceva in Unresectable NSCLC; SWOG = South West Oncology Group.

Six trials (2672 patients; 99% of total randomized) reported PFS for all patients irrespective of EGFR mutation status. The metaregression suggested that treatment effect varied according to the proportion of patients with wild type EGFR (*P* = .11). When 100% of patients had wild type EGFR, the model suggested that there is no difference in PFS with TKIs compared with no active treatment (HR, 0.95; 95% CI, 0.65-1.38; *P* = .78), whereas when 100% of patients had EGFR mutations, a large benefit of TKIs was indicated (HR, 0.12; 95% CI, 0.02-0.66; *P* = .015; Figure 5).^38-43^ However, the metaregression was based on only 6 trials and was clearly limited.

### Interaction Between Treatment Effect and Histology in Patients With Wild Type EGFR

We conducted an exploratory analysis to assess whether the benefit of TKIs in patients with wild type EGFR was related to histological type (adenocarcinoma/squamous cell carcinoma). Data were available for 4 trials and 2129 patients (1430 adenocarcinoma; 699 squamous/other nonadenocarcinoma). There was a significant difference in effect between the 2 subgroups (interaction HR, 1.41; 95% CI, 1.11-1.80; *P* = .004) with little suggestion of variation between trials (heterogeneity *P* = .347; *I*^2^, 3.8%). However, benefits of TKI were observed for patients with squamous (HR, 0.77; 95% CI, 0.64-0.92; *P* = .004; *I*^2^, 0%; heterogeneity *P* = .89) and adenocarcinoma (HR, 0.59; 95% CI, 0.52-0.66; *P* < .0001; *I*^2^, 79%; heterogeneity *P* = .002).

## Overall Survival

Three trials reported OS according to mutation status. We found no evidence to suggest a difference in the effect of TKIs in patients with mutations compared with those with wild type disease (interaction HR, 1.40; 95% CI, 0.76-2.57; *P* = .28; Table 2). This relationship was similar between the trials (heterogeneity *P* = .49; *I*^2^, 0%).

## Discussion

Taken together, evidence from 3 distinct analytical approaches suggests a difference in the effect of TKIs on PFS according to EGFR mutation status. For patients with wild type EGFR, TKIs seem to be an ineffective second-line treatment compared with chemotherapy, but might be effective as maintenance treatment, compared with no active treatment. In both settings, TKIs offer PFS benefits to patients with mutated EGFR.

Pooling the estimated interactions between treatment effects and patient characteristics for each trial, using only within-trial information and avoiding ecological bias,^14^ provides the most reliable estimate of the relationship between the effect of TKIs and EGFR mutation status. Nevertheless, it relies on trials reporting results for patients with wild type EGFR and EGFR mutations separately. Although not all trials tested patients systematically, we have no reason to suspect selective reporting of results or selective testing of patients, which could introduce bias. However, the precision of the estimates of interaction was inevitably limited by the relatively low numbers of included trials reporting results for both mutation subgroups (trials of second line treatment, n = 9, and in the maintenance setting, n = 4).

Incorporating additional data, mainly from trials that recruited only patients with wild type EGFR, enabled us to provide further evidence that TKIs are inferior to chemotherapy as second-line treatment in patients with wild type EGFR, reducing median PFS by approximately 3 weeks. As maintenance treatment, we found that TKIs offer a modest improvement in median PFS compared with no active treatment of approximately 3 weeks for patients with wild type EGFR. We did, however, see some evidence of inconsistency between the trial results in the second-line treatment setting, which might reflect the clinically heterogeneous nature of this large group of patients. There will of course be variation in terms of known prognostic factors such as age, tumor histology, and stage, however, it is also possible that other, as yet undefined characteristics, might further explain this variability. Although pooling results for patient subgroups from trials in this way might introduce bias^14^ and the meta-analysis is again limited because many trials tested only a relatively small subset of patients for mutation status, or did not test at all, they do provide the best available estimate of the effects of TKIs in patients with wild type and mutated EGFR.

Using metaregression, including data from all randomized patients from all eligible trials, we assessed how the proportion of patients with wild type EGFR modified the effect of TKIs on PFS. The results suggest that the benefit of TKIs relative to chemotherapy on PFS diminishes with an increasing proportion of patients with wild type EGFR. This pattern holds for the maintenance setting, suggesting no evidence of a benefit of TKIs when 100% of patients have wild type EGFR. These metaregressions relied in part on assumptions about the EGFR mutation status of trial populations. Furthermore, the relationship between the effect of TKIs and the proportion of patients with wild type EGFR might not be representative of the true relationship between the effect of TKIs and the mutation status of individual patients.^14,45^ There are clearly limitations to this approach, not only that we had to make assumptions or estimates of the proportions of patients with wild type EGFR for a number of the trials. The relatively small number of data points (second-line setting, n = 12; maintenance setting, n = 6) and that in part because of estimates made, the proportions tend to cluster around either the 60% or 90% positions, mean that the regression model is limited. Nonetheless, this approach has allowed us to maximize the available data to include all relevant trials, and the results do add further support for our other analyses. Although none of the individual approaches is without potential limitations, using results obtained from 3 distinct methods has increased our confidence in interpreting and drawing conclusions regarding the effect of EGFR mutation status on the response to erlotinib and gefitinib in these 2 treatment settings.

These systematic reviews are the first to have estimated the interaction between the effects of TKIs on PFS in patients with wild type EGFR and those with EGFR mutations appropriately and reliably.^14^ Furthermore, they are the first to have based interpretation on a range of analyses, making the best use of all available data and to assess consistency of results. We have attempted to evaluate the effect of treatment in wild type patients, who form the majority of patients with NSCLC worldwide. Since the importance of activating EGFR mutations in patients' response to TKIs were recognized, research has concentrated on patients carrying such mutations. Moreover, a recent review focused on the effect of TKIs in mutation-positive patients and only used the limited reported data from patients who were tested for EGFR mutations.^12^ Using or estimating the proportions of patients with wild type EGFR, we included results from all trials in a metaregression, rather than only the minority of patients for whom results according to mutational status were reported. We avoided estimating effects based on all randomized patients irrespective of EGFR status, which are potentially misleading.

Although we provided clear evidence of a difference in the effect of TKIs according to mutation status on PFS, there was no evidence to support a difference in their effect on OS in either treatment setting. However, most of the included trials allowed treatment crossover on progression, inevitably making the OS results more difficult to interpret. The full effect of TKI treatment on OS in patients with advanced NSCLC therefore remains unclear.

In the maintenance setting, our strict eligibility criteria meant that we included only trials in which treatment comparisons were unconfounded. This inevitably limited the number of trials and patients available for this analysis and hence our results must be viewed with particular caution. Furthermore, the comparator in these trials was essentially no active treatment. It is unclear whether the potential benefit of TKIs for patients with wild type EGFR would persist if compared for example, with pemetrexed (for patients with adenocarcinoma) as is recommended in clinical guidelines^6-7^ and NICE TA162, NCCN. We did not find any trials that directly compared TKIs with chemotherapy in the maintenance setting. One 3-arm trial included in this meta-analysis compared each of gemcitabine and erlotinib with observation alone^39^ but no comparison between the 2 treatments was reported.

Currently TKIs and chemotherapy are recommended options for second-line treatment for patients with wild type EGFR. Our results bring this into doubt and suggest that for patients with wild type EGFR who are well enough to receive it, chemotherapy should be viewed as the standard of care. Furthermore, particularly outside of East Asia, most unselected patients will have wild type EGFR. Guidelines that recommend TKIs for unselected populations should be reconsidered. Our results highlight the importance of suitable biopsies and reliable EGFR mutation testing to guide optimal clinical treatment.

Our findings regarding maintenance treatment, coupled with our results in the second-line setting might lead us to surmise that, compared with appropriate chemotherapy, patients with wild-type EGFR are unlikely to benefit from TKIs. However, for patients in whom no alternative is recommended, for example, in patients with squamous cell carcinoma, TKIs might be considered. Without direct comparisons of TKIs with chemotherapy in this setting, the best treatment options remain unclear.

## Conclusion

There is still uncertainty regarding the best treatment option for the overwhelming majority of advanced NSCLC patients worldwide with wild type EGFR. However, based on these results, TKIs are not an appropriate second-line treatment for patients who are fit to receive chemotherapy, but might offer some scope as maintenance treatment.

## Disclosure

The authors have stated that they have no conflicts of interest.

## Figures and Tables

**Supplemental Table 1:** Assessment of Risk of Bias for Included Trials

Trial	Sequence Generation	Allocation Concealment	Blinding of Participants/Personnel	Blinding of Outcome Assessment^a^	Incomplete Outcome Data Addressed	Free of Selective Reporting
**Second-Line Treatment**						
SIGN^26^ Full publication	Low risk	Low risk	NA	NA	Low risk	Low risk
	Randomized 1:1 to 1 of 2 treatment groups	Randomization was performed at the site using sealed randomization envelopes which were allocated sequentially to patients			All randomized patients are included in the analysis	Reports PFS and OS in full
V-15-92^27^ Full publication	Low risk	Unclear	NA	NA	Low risk	Low risk
	Patients were randomly assigned using stratification factors of sex, PS, histology, and study site	NR			490 Patients randomized; 1 patient excluded due to protocol violation; 489 patients ITT	Reports OS in ITT patients and PFS for ‘assessable for response’ population
Herbst et al^28^ Full publication	Low risk	Unclear	NA	NA	Low risk	Low risk
	Patients were randomly assigned on a 1:1:1 basis	NR			The analyses included all treated patients	Reports PFS and OS in full
INTEREST^29^ Full publication	Low risk	Low risk	NA	NA	Low risk	Low risk
	Patients were randomly assigned with dynamic balancing	Used a centralized registration and randomization center, contacted by telephone, to assign patients to a specific treatment group			1466 Patients randomized; 1433 patients per protocol population (OS); 1316 patients evaluable for response population (PFS)	Reports PFS in evaluable for response population (89% of randomized patients) and OS in per protocol population (97% of randomized patients)
ISTANA^30^ Full publication	Low risk	Unclear	NA	NA	Low risk	Low risk
	Eligible patients were randomly assigned to receive gefitinib or docetaxel after stratification for histology, sex, PS, best response to previous therapy, smoking history, and participating center	NR			All randomized patients are included in the analysis	Reports PFS and OS in full
TITAN^31^ Full publication	Low risk	Low risk	NA	NA	Low risk	Low risk
	Patients who were enrolled into TITAN were randomly assigned (1:1) using an adaptive randomization method (minimization as proposed by Pocock and Simon^46^)	Randomization and stratification instructions were obtained through a third-party telephone interactive voice response system			All randomized patients are included in the analysis	Reports PFS and OS in full
HORG^32^ Full publication	Low risk	Low risk	NA	NA	Low risk	Low risk
	Patients were centrally randomized by computer at a 1:1 ratio and stratified according to PS, disease stage, age, and response to first-line treatment	Patients were centrally randomized using a computer			357 Patients randomized; 332 patients (93%) received treatment and were analyzed	Reports time to progression and OS in full
CTONG 0806^9^ Abstract and slides	Low risk	Low risk	NA	NA	Low risk	Low risk
	Patients were randomized to receive gefitinib orally or pemetrexed	Centrally randomized			161 Patients randomized; 157 (98%) were evaluable	Reports PFS and OS in full
TAILOR^8^ Full publication	Low risk	Low risk	NA	NA	Low risk	Low risk
	Treatment was randomly allocated in a 1:1 ratio using a minimization algorithm	A customized, Web-based database was set up for registration, randomization, monitoring, local data entry, and central data management			222 Patients randomized; 219 (99%) patients included in ITT analyses	Reports PFS and OS in full
KCSG-LU08-01^33^ Full publication	Low risk	Low risk	NA	NA	Low risk	Low risk
	Patient randomization was stratified according to ECOG PS or sex	Patients were consecutively assigned according to a predefined computer-generated randomization scheme			141 Patients randomized; 135 (96%) patients received treatment and were analyzed	Reports OS and PFS in full
PROSE^34^ Full publication	Low risk	Low risk	NA	NA	Low risk	Low risk
	Treatment was randomly allocated using a minimization algorithm, which stratified treatment allocation according to smoking history (never, former, or current smokers), ECOG PS (0-1, or 2), proteomic test classification (poor or good), and center	Centrally randomized			285 Patients randomized; 22 patients excluded for protocol violations or due to not receiving treatment; 263 (92%) patients were analyzed	Reports OS and PFS in full
DELTA^35^ Full publication	Low risk	Low risk	NA	NA	Low risk	Low risk
	Eligible patients were randomly assigned 1:1 to erlotinib or docetaxel using the minimization method according to sex, PS, histology, and institution	Centrally randomized			All randomized patients are included in the analysis	Reports OS and PFS in full
Li et al^37^ Full publication	Low risk	Low risk	NA	NA	Low risk	Low risk
	Patients were randomly assigned in a 1:1 ratio; randomization was stratified according to sex, PS, and smoking history using a minimization algorithm	Centrally randomized			All randomized patients are included in the analysis	Reports OS and PFS in full
*Li et al^36^ Full publication	Unclear	Unclear	Unclear	Unclear	Unclear	Unclear
**Maintenance Treatment**						
SATURN^38^ Full publication	Low risk	Low risk	Low risk	Low risk	Low risk	Low risk
	Randomization was done using a 1:1 adaptive method using minimization as proposed by Pocock and Simon^46^	Via a third-party voice response system	Allocation was not blinded but is unlikely to bias the outcome of PFS	The randomization list was not made available to the study centers, trial monitors, statisticians, or study sponsor	5 Patients who progressed before randomization were not included in the analysis of PFS (1/438 erlotinib; 4/451 placebo)	Reports PFS and OS in full
EORTC 08021^40^ Full publication	Low risk	Low risk	Low risk	Low risk	Low risk	Low risk
	Minimization with stratification according to stage, PS, best response, and institution	Centralized double blind random assignment	Double-blind	Unblinding was only allowed if the investigator needed the information to determine subsequent therapy	All randomized patients are included in the analysis	Reports PFS and OS in full
IFCT-GFPC-0502^39^ (NCT00300586) Full publication	Low risk	Low risk	Low risk	Low risk	Low risk	Low risk
	Minimization adaptive randomization, stratified according to center, sex, histology, smoking status, and response to first-line CT	Randomization was computerized and centrally located	Lack of blinding is unlikely to bias the outcome of PFS	Disease progression was reviewed by a panel of investigators who were blinded to randomization, independently of the treating investigator	All randomized patients were included in the analysis	Reports PFS and OS in full
ATLAS^43^ Full publication	Low risk	Low risk	Low risk	Low risk	Low risk	Low risk
	Patients were randomized (1:1), and stratified according to initial chemotherapy choice, smoking status, and ECOG PS	Centrally randomized via an interactive voice response system	The study sponsor, study investigators, and patients were blinded to treatment	A data safety monitoring committee monitored safety and efficacy	All randomized patients were included	Reports PFS and OS in full
SWOG S0023^42^ Full publication	Unclear	Unclear	Low risk	Low risk	Low risk	Low risk
	Randomly assigned	Not reported	No details reported/not blinded but lack of blinding is unlikely to bias the outcome of PFS	No details reported/not blinded but lack of blinding is unlikely to bias the outcome of PFS	243 Randomly assigned and all included in analyses. An additional 18 patients (7%) who were randomly assigned were found to be ineligible – they were not analyzed	Reports PFS and OS in full
INFORM^41^ Full publication	Low risk	Low risk	Low risk	Low risk	Low risk	Low risk
	The system generated randomization codes based on dynamic balancing algorithms wrt histology and smoking history	Randomization was done centrally by a third party randomization center that had no other role in the study	Active and placebo drugs were identical in form and packaging to ensure blinding. Further steps to ensure blinding included blinding of AstraZeneca personnel	Investigators and participants were blinded to study treatment until the primary analysis of PFS was complete	All randomized patients appear to have been included in the analyses	Reports PFS and OS in full

Abbreviations: ATLAS = Avastin Tarceva Lung Adenocarcinoma Study; CT = Chemotherapy; CTONG = Chinese Thoracic Oncology Group; DELTA = Docetaxel and Erlotinib Lung Cancer Trial; ECOG = Eastern Cooperative Oncology Group; EORTC = European Organisation for the Research and Treatment of Cancer; HORG = Hellenic Oncology Research Group; IFCT-GFPC = Partenariat Intergroupe Francophone de Cancérologie Thoracique - Groupe Français de Pneumo-Cancérologie; INFORM = Iressa in NSCLC FOR Maintenance; INTEREST = IRESSA Non-small-cell lung cancer Trial Evaluating REsponse and Survival against Taxotere; ISTANA = Iressa as Second-line Therapy in Advanced NSCLC; ITT = Intention to treat; KCSG = Korean Cancer Study Group; OS = overall survival; PFS = progression-free survival; PROSE =Predicting Response to Second-Line Therapy Using Erlotinib; PS = performance status; SATURN = Sequential Tarceva in Unresectable NSCLC; SIGN = Second-line Indication of Gefitinib in NSCLC; SWOG = South West Oncology Group; TAILOR = Tarceva Italian Lung Optimization Trial; TITAN = Tarceva In Treatment of Advanced NSCLC; wrt = with respect to.

∗ Li trial published in Chinese language.

a Blinding only possible for trials comparing TKI with placebo in the maintenance setting.

**Table 1:** Trial and Patient Characteristics (Based on All Randomized Patients)

Trial	Accrual Period	Patient n	TKI	Control	Median Age (Range)	Sex (% Female)	PS (% 0/1)	Ethnicity	Smoking History (% Never)	Histology (% Adenocarinoma)	Patients With Known EGFR Status (% of Total Randomized)	EGFR Mutation, n (% of Total With Known Status)	EGFR Wild Type, n (% of Total With Known Status)
**Trials of Second-Line Treatment**													
SIGN^26^	2003-2004	141	Gefitinib	Docetaxel	61 (29-85)	30	67	Western	25	Unknown	NR	NR	NR
V-15-32^27^	2003-2006	489 (387^a^)	Gefitinib	Docetaxel	Unknown	38	96	Asian	32	78	57 (12)	31 (55)	26 (45)
Herbst et al^28^	2004-2005	79	Erlotinib	Docetaxel or pemetrexed with bevacizumab	65.5 (40-88)	49	100	Western	13	78	30 (38)	1 (3)	29 (97)
INTEREST^29^	2004-2006	1466 (1316^a^)	Gefitinib	Docetaxel	60.5 (20-84)	35	88	Western	20	54	267 (18)	38 (14)	229 (86)
ISTANA^30^	2005-2006	161	Gefitinib	Docetaxel	57.5 (20-74)	38	93	Asian	41	68	NR	NR	NR
Li et al^36^	2006-2008	98	Gefitinib	Docetaxel	Unknown	Unknown	Unknown	Asian	Unknown	Unknown	NR	NR	NR
TITAN^31^	2006-2010	424	Erlotinib	Docetaxel or pemetrexed	59 (22-79)	24	80	Western	17	50	160 (38)	11 (7)	149 (93)
HORG^32^	2006-2010	332	Erlotinib	Pemetrexed	65.5 (37-86)	18	85	Western	16	77 (non-sq)	NR	NR	NR
CTONG 0806^9^^,^^b^	2009-2012	157	Gefitinib	Pemetrexed	56.5 (24-78)	36	100	Asian	49	96	157 (100)	Only WT patients	157 (100)
TAILOR^8^^,^^b^	2007-2012	219	Erlotinib	Docetaxel	66.5 (35-83)	31	91	Western	22	68 (greater % in TKI arm)	219 (100)	Only WT patients	219 (100)
KCSG-LU08-01^33^	2008-2010	135	Gefitinib	Pemetrexed	61 (30-78) (younger in TKI arm)	85	91	Western	100	100	71 (53)	33 (46)	38 (54)
PROSE^34^	2008-2012	263	Erlotinib	Docetaxel or pemetrexed	65 (33-85)	27	94	Western	14	88 (non-sq)	177 (67)	14 (8)	163 (92)
DELTA^35^	2009-2012	301	Erlotinib	Docetaxel	67.5 (31-85)	29	96	Asian	25	69	255	51 (20)	199 (78)
Li et al^37^^,^^b^	2008-2014	123	Erlotinib	Pemetrexed	54.5 (30-75)	36	94	Asian	26	100	123 (100)	Only WT patients	123 (100)
Total		4388 (4136)									1516 (35)	179 (12)	1332 (88)
**Trials of Maintenance Treatment**													
SATURN^38^	2005-2008	889	Erlotinib	Placebo	60 (30-83)	26	100%	Western	17	45	368 (41)	40 (11)	328 (89)
IFCT-GFPC 0502 (NCT00300586)^39^	2006-2009	310^c^	Erlotinib	Observation	58 (36-72)	27	100%	Western	9	65	114 (37)	8 (7)	106 (93)
EORTC 08021^40^	2004-2009	173	Gefitinib	Placebo	61 (28-80)	23	94%	Western	22	51	NR	NR	NR
INFORM^41^	2008-2009	296	Gefitinib	Placebo	55 (20-75)	41	98%	Asian	54	71	79 (27)	30 (38)	49 (62)
SWOG S0023^42^	2001-2005	261	Gefitinib	Placebo	61 (24-81)	37	96%	Western	Unknown	31	NR	NR	NR
ATLAS^43^^,^^d^	2005-2008	768	Erlotinib	Placebo	64 (range unknown)	48	100%	Western	16	81	347 (45)^e^	52 (15)	295 (85)
Total		2697									908 (34)	130 (14)	778 (86)

Abbreviations: ATLAS = Avastin Tarceva Lung Adenocarcinoma Study; CTONG = Chinese Thoracic Oncology Group; DELTA = Docetaxel and Erlotinib Lung Cancer Trial; EGFR = epidermal growth factor receptor; EORTC = European Organisation for Research and Treatment of Cancer; HORG = Hellenic Oncology Research Group; IFCT-GFPC = Partenariat Intergroupe Francophone de Cancérologie Thoracique-Groupe Français de Pneumo-Cancérologie; INFORM = Iressa in NSCLC FOR Maintenance; INTEREST = IRESSA Non-small-cell lung cancer Trial Evaluating REsponse and Survival against Taxotere; ISTANA = Iressa as Second-line Therapy in Advanced NSCLC; KCSG = Korean Cancer Study Group; non-sq = Non-Squamous; PROSE = Predicting Response to Second-Line Therapy Using Erlotinib; PS = performance status; SATURN = Sequential Tarceva in Unresectable NSCLC; SIGN = Second-line Indication of Gefitinib in NSCLC; SWOG = South West Oncology Group; TAILOR = Tarceva Italian Lung Optimization Trial; TITAN = Tarceva In Treatment of Advanced NSCLC; TKI = tyrosine kinase inhibitor; WT = wild type.

a Progression-free survival analyses for patient number in parentheses, but patient characteristics reported for all patients.

b Only randomized patients with wild type EGFR.

c Three-arm trial including 464 randomized patients but only 2 arms included here.

d Includes bevacizumab in both arms.

e Total for progression-free survival, total for overall survival is 345.

**Table 2:** Results for Overall Survival

	Trial, n	Patient, n	Fixed Effect			Random Effect			Interaction HR^a^ (95% CI) *P*	Interaction Heterogeneity, *P*
			HR	95% CI	*P*	HR	95% CI	*P*		
**Second-Line Treatment**										
EGFR wild type	9	1400	1.06	0.93-1.22	.37	1.06	0.93-1.20	.37	1.15 (0.60-2.18) .68	.37
EGFR mutations	4	97	0.90	0.49-1.64	.72	0.90	0.49-1.64	.72		
**Maintenance Treatment**										
EGFR wild type	3	707	0.85	0.72-1.02	.06	0.87	0.70-1.07	.70	1.40 (0.76-2.57) .28	.49
EGFR mutations	3	120	0.59	0.33-1.05	.07	0.59	0.33-1.05	.07		

Abbreviations: EGFR = epidermal growth factor receptor; HR = hazard ratio; TKI = tyrosine kinase inhibitor.

a Interaction HR > 1 shows greater TKI benefit for mutated EGFR.

## References

[1] Fukuoka M., Wu Y.L., Thongprasert S. (2011). Biomarker analyses and final overall survival results from a phase III, randomized, open-label, first-line study of gefitinib versus carboplatin/paclitaxel in clinically selected patients with advanced non–small-cell lung cancer in Asia (IPASS). J Clin Oncol.

[2] Rosell R., Carcereny E., Gervais R. (2012). Erlotinib versus standard chemotherapy as first-line treatment for European patients with advanced EGFR mutation-positive non–small-cell lung cancer (EURTAC): a multicentre, open-label, randomised phase 3 trial. Lancet Oncol.

[3] Makoto M., Akira I., Kunihiko K. (2010). Gefitinib or chemotherapy for non–small-cell lung cancer with mutated EGFR. N Engl J Med.

[4] Caicun Z., Yi-Long W., Gongyan C. (2011). Erlotinib versus chemotherapy as first-line treatment for patients with advanced *EGFR* mutation-positive non–small-cell lung cancer (OPTIMAL, CTONG-0802): a multicentre, open-label, randomised, phase 3 study. Lancet Oncol.

[5] Mitsudomi T., Morita S., Yatabe Y. (2010). Gefitinib versus cisplatin plus docetaxel in patients with non–small-cell lung cancer harbouring mutations of the epidermal growth factor receptor (WJTOG3405): an open label, randomised phase 3 trial. Lancet Oncol.

[6] Azzoli C.G., Baker S., Temin S. (2009). American Society of Clinical Oncology Clinical Practice Guideline Update on Chemotherapy for Stage IV Non–Small-Cell Lung Cancer. J Clin Oncol.

[7] Peters S., Adjei A.A., Gridelli C. (2012). Metastatic non–small-cell lung cancer (NSCLC): ESMO Clinical Practice Guidelines for diagnosis, treatment and follow-up. Ann Oncol.

[8] Garassino M.C., Martelli O., Broggini M. (2013). Erlotinib versus docetaxel as second-line treatment of patients with advanced non–small-cell lung cancer and wild-type EGFR tumours (TAILOR): a randomised controlled trial. Lancet Oncol.

[9] Zhou Q., Cheng Y., Zhao M.F. (2013). Final results of CTONG 0806: a phase II trial comparing pemetrexed with gefitinib as second-line treatment of advanced non-squamous NSCLC patients with wild-type EGFR. J Thorac Oncol.

[10] Petrelli F., Borgonovo K., Cabiddu M., Barni S. (2012). Efficacy of EGFR tyrosine kinase inhibitors in patients with EGFR-mutated non–small-cell lung cancer: a meta-analysis of 13 randomized trials. Clin Lung Cancer.

[11] Gao H., Ding X., Wei D. (2011). Efficacy of erlotinib in patients with advanced non–small-cell lung cancer: a pooled analysis of randomized trials. Anticancer Drugs.

[12] Lee C.K., Brown C., Gralla R.J. (2013). Impact of EGFR inhibitor in non–small-cell lung cancer on progression-free and overall survival: a meta-analysis. J Natl Cancer Inst.

[13] Lee J.K., Hahn S., Kim D.W. (2014). Epidermal growth factor receptor tyrosine kinase inhibitors vs conventional chemotherapy in non–small cell lung cancer harboring wild-type epidermal growth factor receptor: a meta-analysis. JAMA.

[14] Fisher D.J., Copas A.J., Tierney J.F., Parmar M.K. (2011). A critical review of methods for the assessment of patient-level interactions in individual patient data (IPD) meta-analysis of randomised trials, and guidance for practitioners. J Clin Epidemiol.

[15] Ellison G., Zhu G., Moulis A. (2013). EGFR mutation testing in lung cancer: a review of available methods and their use for analysis of tumour tissue and cytology samples. J Clin Pathol.

[16] Higgins J.P., Green S.J. (2011). Cochrane Handbook for Systematic Reviews of Interventions. *The Cochrane Collaboration*.

[17] Higgins J.P., Altman D.G., Gotzsche P.C. (2011). The Cochrane Collaboration's tool for assessing risk of bias in randomised trials. BMJ.

[18] Parmar M.K., Torri V., Stewart L.A. (1998). Extracting summary statistics to perform meta-analyses of the published literature for survival endpoints. Stat Med.

[19] Williamson P.R., Tudur Smith C., Hutton J.L., Marson A.G. (2002). Aggregate data meta-analysis with time-to-event outcomes. Stat Med.

[20] Tierney J.F., Stewart L.A., Ghersi D., Burdett S., Sydes M.R. (2007). Practical methods for incorporating summary time-to-event data into meta-analysis. Trials.

[21] Higgins J.P., Thompson S.G., Deeks J.J., Altman D.G. (2003). Measuring inconsistency in meta-analyses. BMJ.

[22] The International Collaborative Ovarian Neoplasm (ICON) Group (2002). Paclitaxel plus carboplatin versus standard chemotherapy with either single-agent carboplatin or cyclophosphomide, doxorubicin, and cisplatin in women with ovarian cancer: the ICON3 randomised trial. Lancet.

[23] Bhatnagar A.R., Singh D.P., Sharma R., Kumbhaj P. (2012). Docetaxel versus gefitinib in patients with locally advanced or metastatic NSCLC pre-treated with platinum-based chemotherapy. J Thorac Oncol.

[24] Dilts D.M., Adjei A.A., Mandrekar S.J. (2010). Impact of trial development time on accruals at CCOPs: the case of the MARVEL trial. J Clin Oncol.

[25] Soria J.C., Barlesi F., Besse B. (2013). Results of the prospective, randomized, and customized NSCLC adjuvant phase II trial (IFCT-0801, TASTE trial) from the French Collaborative Intergroup (abstract 7505). J Clin Oncol.

[26] Cufer T., Vrdoljak E., Gaafar R. (2006). Phase II, open-label, randomized study (SIGN) of single-agent gefitinib (IRESSA) or docetaxel as second-line therapy in patients with advanced (stage IIIb or IV) non–small-cell lung cancer. Anticancer Drugs.

[27] Maruyama R., Nishiwaki Y., Tamura T. (2008). Phase III study, V-15-32, of gefitinib versus docetaxel in previously treated Japanese patients with non–small-cell lung cancer. J Clin Oncol.

[28] Herbst R.S., O'Neill V.J., Fehrenbacher L. (2007). Phase II study of efficacy and safety of bevacizumab in combination with chemotherapy or erlotinib compared with chemotherapy alone for treatment of recurrent or refractory non–small-cell lung cancer. J Clin Oncol.

[29] Douillard J.Y., Shepherd F.A., Hirsh V. (2010). Molecular predictors of outcome with gefitinib and docetaxel in previously treated non–small-cell lung cancer: data from the randomized phase III INTEREST trial. J Clin Oncol.

[30] Lee D.H., Park K., Kim J.H. (2010). Randomized phase III trial of gefitinib versus docetaxel in non–small-cell lung cancer patients who have previously received platinum-based chemotherapy. Clin Cancer Res.

[31] Ciuleanu T., Stelmakh L., Cicenas S. (2012). Efficacy and safety of erlotinib versus chemotherapy in second-line treatment of patients with advanced, non–small-cell lung cancer with poor prognosis (TITAN): a randomised multicentre, open-label, phase 3 study. Lancet Oncol.

[32] Karampeazis A., Voutsina A., Souglakos J. (2013). Pemetrexed versus erlotinib in pretreated patients with advanced non–small-cell lung cancer: a Hellenic Oncology Research Group (HORG) randomized phase 3 study. Cancer.

[33] Ahn M.J., Sun J.M., Kim S.W. (2011). Randomized phase III trial of gefitinib or pemetrexed as second line treatment in patients with non–small-cell lung cancer previously treated with platinum-based chemotherapy (KCSG-LU08-01). J Thorac Oncol.

[34] Gregorc V., Novello S., Lazzari C. (2014). Predictive value of a proteomic signature in patients with non–small-cell lung cancer treated with second-line erlotinib or chemotherpay (PROSE): a biomarker-statified, randomised phase 3 trial. Lancet Oncol.

[35] Kawaguchi T., Ando M., Asami K. (2014). Randomised phase III trial of erlotinib versus docetaxel as second or third-line therapy in patients with advanced non–small-cell lung cancer: docetaxel and erlotinib lung cancer trial (DELTA). J Clin Oncol.

[36] Li H., Wang X., Hua F. (2010). Second-line treatment with gefitinib or docetaxel for advanced non–small-cell lung cancer [in Chinese]. Chin J Clin Oncol.

[37] Li N., Ou W., Yang H. (2014). A randomized phase 2 trial of erlotinib versus pemetrexed as second-line therapy in the treatment of patients with advanced EGFR wild-type and EGFR FISH-positive lung adenocarcinoma. Cancer.

[38] Cappuzzo F., Ciuleanu T., Stelmakh L. (2010). Erlotinib as maintenance treatment in advanced non–small-cell lung cancer: a multicentre, randomised, placebo-controlled phase 3 study. Lancet Oncol.

[39] Perol M., Chouaid C., Perol D. (2012). Randomized, phase III study of gemcitabine or erlotinib maintenance therapy versus observation, with predefined second-line treatment, after cisplatin-gemcitabine induction chemotherapy in advanced non–small-cell lung cancer. J Clin Oncol.

[40] Gaafar R.M., Surmont V.F., Scagliotti G.V. (2011). A double-blind, randomised, placebo-controlled phase III intergroup study of gefitinib in patients with advanced NSCLC, non-progressing after first line platinum-based chemotherapy (EORTC 08021/ILCP 01/03). Eur J Cancer.

[41] Zhang L., Ma S., Song X. (2012). Gefitinib versus placebo as maintenance therapy in patients with locally advanced or metastatic non–small-cell lung cancer (INFORM; C-TONG 0804): a multicentre, double blind randomised phase 3 trial. Lancet Oncol.

[42] Kelly K., Chansky K., Gaspar L.E. (2008). Phase III trial of maintenance gefitinib or placebo after concurrent chemoradiotherapy and docetaxel consolidation in inoperable stage III non–small-cell lung cancer: SWOG S0023. J Clin Oncol.

[43] Kabbinavar F., Fehrenbacher L., Hainsworth J. (2014). Biomarker analyses from a randomized, placebo-controlled, phase IIIb trial comparing bevacizumab with our without erlotinib as maintenance therapy for treatment of advanced non–small-cell lung cancer (ATLAS). J Thorac Oncol.

[44] Vale C.L., Tierney J.F., Burdett S. (2013). Can trial quality be reliably assessed from published reports of cancer trials: evaluation of risk of bias assessments in systematic reviews. BMJ.

[45] Berlin J.A., Santanna J., Schmid C.H. (2002). Individual patient- versus group-level data meta-regressions for the investigation of treatment effect modifiers: ecological bias rears its ugly head. Stat Med.

[46] Pocock S.J., Simon R. (1975). Sequential treatment assignment with balancing for prognostic factors in the controlled clinical trial. Biometrics.

